# Utilizing the Potential of Antimicrobial Peptide LL-37 for Combating SARS-COV- 2 Viral Load in Saliva: an *In Silico* Analysis

**DOI:** 10.1055/s-0041-1739444

**Published:** 2021-12-22

**Authors:** Nireeksha Nireeksha, Pavan Gollapalli, Sudhir Rama Varma, Mithra N. Hegde, N. Suchetha Kumari

**Affiliations:** 1 Department of Conservative Dentistry and Endodontics, AB Shetty Memorial Institute of Dental Sciences, NITTE (deemed to be) University, Deralakatte, Mangaluru, Karnataka, India; 2 Central Research Laboratory, K.S. Hegde Medical Academy, NITTE (deemed to be) University, Deralakatte, Mangaluru, Karnataka, India; 3 Department of Clinical Sciences, Ajman University, Ajman, United Arab Emirates; 4 Centre of Medical and Bio-Allied Health Sciences Research, Ajman University, Ajman, United Arab Emirates; 5 Department of Biochemistry, K.S. Hegde Medical Academy, NITTE (deemed to be) University, Deralakatte, Mangaluru, Karnataka, India

**Keywords:** *in silico* analysis, LL-37, antimicrobial peptide, SARS-CoV-2, COVID-19, saliva

## Abstract

Limiting the spread of virus during the recent pandemic outbreak was a major challenge. Viral loads in saliva, nasopharyngeal and oropharyngeal swabs were the major cause for droplet transmission and aerosols. Saliva being the major contributor for the presence of viral load is the major key factor; various mouthwashes and their combination were analyzed and utilized in health care centers to hamper the spread of virus and decrease viral load. The compositions of these mouthwashes to an extent affected the viral load and thereby transmission, but there is always a scope for other protocols which may provide better results. Here we evaluated the potential of antimicrobial peptide LL-37 in decreasing the viral load of severe acute respiratory syndrome coronavirus 2 (SARS-CoV-2) through an *in silico* work and evidence from other studies. This narrative review highlighted a brief nonsystematic methodology to include the selected articles for discussion. Accessible electronic databases (Medline, Scopus, Web of Science, SciELO, and PubMed) were used to find studies that reported the salivary viral load of SARS-CoV-2 published between December 2019 and June 2021. The following keywords were utilized for brief searching of the databases: “saliva,” “viral load,” and “SARS-CoV-2.” Articles in English language, *in vitro* cell-line studies, *ex vivo* studies, and clinical trials explaining the viral load of SARS-CoV-2 in saliva and strategies to decrease viral load were included in this review. The search was complemented by manual searching of the reference lists of included articles and performing a citation search for any additional reviews. The antiviral potential of cationic host defense peptide LL-37 was evaluated using computational approaches providing *in silico* evidence. The analysis of docking studies and the display of positive interfacial hydrophobicity of LL-37 resulting in disruption of COVID-19 viral membrane elucidate the fact that LL-37 could be effective against all variants of SARS-CoV-2. Further experimental studies would be needed to confirm the binding of the receptor-binding domain with LL-37. The possibility of using it in many forms further to decrease the viral load by disrupting the viral membrane is seen.

## Introduction

The emergence of the novel coronavirus has received worldwide attention due to its high transmission and reproduction rate (∼2.2 ≤ [1.4–6.5]). Coronaviruses are enveloped RNA viruses, and two strains of them—severe acute respiratory syndrome coronavirus (SARS-CoV) and Middle East respiratory syndrome coronavirus (MERS-CoV)—are zoonotic in origin and known to cause fatal respiratory diseases and the recent SARS-CoV-2 is responsible for the global pandemic. The viral infection turned into a devastating pandemic condition due to human-to-human transmission across the globe. The early detection of viral RNA in saliva peaks during the onset of symptoms. The attachment of SARS-CoV-2 to the ACE2 receptor on host cells of tongue and salivary gland makes saliva a major candidate for transmission of this virus via droplet or aerosol transmission, further contributing to the disease transmission. 1 2 Post outbreak of the pandemic, various guidelines for treatment strategies have been formulated by the Ministry of Health and Family Welfare, Government of India to prevent transmission in medical dispensaries, hospitals, and dental clinics. They mainly concentrated on various ways which can prevent the transmission, such as using personal protective equipment, N95 masks, face shields, triple-layer masks, and single ventilated rooms for aerosol-related procedures. Even though these protocols are implemented, they do not completely mitigate the risk of transmission of the coronavirus 2019 (COVID-19) virus.

The key issue during this period was how to prevent transmission, which could decrease the requirement of treatment. Various reports and editorials mentioned the risk of health care workers (HCWs) and clinic setups. 3 The risk of acquiring infection among HCWs including doctors, nurses, ward boys, and dentists who are exposed to these individuals further become a source of infection to other individuals who are not yet infected or are in isolation. Prevention of infectious diseases in HCWs serves three purposes: the health of the HCW, the prevention of work restrictions, and the reduction of hospital-acquired infections. In China around 3,300 HCWs were infected and 22 died due to the COVID-19 virus outbreak. 3 The major concern was the transmission of droplets and aerosols. Basically, various clinics implemented use of HEPA filters, ultraviolet lights, fumigation and antifogging device, and high suction devices in COVID wards and clinics where risk of transmission still exists. Identifying measures to restrict transmission from the source is still a challenge. There have been various agents which are being utilized and discussed for reducing the viral load in saliva. In our review we would like to discuss the potential role of human antimicrobial peptide (AMP) LL-37 in mitigating the risk instilled by COVID-19 viral load in saliva.

AMP LL-37 is a human antimicrobial protein of 18 kDa, only known member of cathelicidin in humans. They are pre-propeptides, with immunomodulatory effect, and are also one among the three defense lines of human body, i.e., the humoral immunity. They possess antimicrobial, antibacterial, and antiviral potential that disrupts pathogen membrane and facilitates wound healing through epidermal growth factors. 4 The antiviral potential of LL-37 has been studied in various viral infections through *in vitro* and animal models. LL-37 primarily interacts with the outer envelope of the virus further after disruption of the membrane, and it irreversibly binds to the DNA and RNA. 5 LL-37 counters the virus in the respiratory epithelium that is secreted from neutrophils, macrophages, and epithelial cells. LL-37 is a potent peptide against various RNA and DNA viruses like Venezuelan equine encephalitis virus (VEEV), Vaccinia virus, Zika virus (ZIKV), hepatitis virus, human rhinovirus (HRV), respiratory syncytial virus (RSV), dengue virus, and human immunodeficiency virus (HIV) in various *in vitro* and animal studies. 6 The mechanism of action discussed in these studies are direct alteration of viral architecture, thereby inactivating virus permanently, robust reduction of viral RNA copies, removal of outer membrane of virus by rapid disintegration, removal of outer membrane, exposure of the virus to adaptive immune system, and inactivation by flocculation and aggregation of negatively charged viral particles that is associated with biomass clumping of these positively charged elements leading to immobilization. This flocculation and aggregation lead to entry prevention of viruses. 7

Innate immune defense may involve synergies between multiple cationic host defense peptides (CHDPs) acting via different mechanisms. CHDPs' responses against viral infection may have therapeutic potential with broader applicability against SARS-CoV-2 and may minimize the potential to promote the emergence of resistant strains by avoiding direct peptide targeting of the virus. Therefore, targeting upregulation of endogenous CHDP expression may be a prophylactic antiviral property with less chances of emergence of resistant strains. 8

## Methods

### Search Strategy and Criteria

This review highlights a brief nonsystematic methodology to include the selected articles for discussion. Accessible electronic databases (Medline, Scopus, Web of Science, SciELO, and PubMed) were used to find studies that reported the salivary viral load of SARS-CoV-2 published between December 2019 and June 2021. The following keywords were utilized for brief searching of the databases: “saliva,” “viral load,” and “SARS-CoV-2.” Articles in English language, *in vitro* cell-line studies, *ex vivo* studies, and clinical trials explaining the viral load of SARS-CoV-2 in saliva and strategies to decrease viral load were included in this review. The search was complemented by manual searching of the reference lists of included articles and performing a citation search for any additional reviews. The number of articles that focused on strategies for reducing viral load was six. The articles which did not provide any information regarding reducing viral load of SARS-CoV-2 and merely discussed the presence of viral load in saliva were excluded. The selected articles are briefly explained in Table 2 .

### Evaluating the Antiviral Potential of Cationic Host Defense Peptide LL-37 through Computational Approaches

One of the most important steps toward rational design and development of therapeutics of LL-37 is inspired by the function and mode of action. Several reports are there demonstrating the antiviral potentiality of the LL-37 peptide. To investigate the peptide–protein interactions of LL-37 with the SARS-CoV-2 targets, we implemented the structural bioinformatics approach ( Table 1 ). For this, approximately 29 potential SARS-CoV-2 target proteins were selected, such as spike RBD (receptor-binding domain), spike monomer (close), spike monomer (open), spike trimer (close), spike trimer (open), S2 (postfusion state), ACE2, membrane protein, E protein, N protein, N protein (C domain), N protein (N domain), main protease, papain-like protease (dimer), papain-like protease (monomer), NSP12 (SARS-CoV-2 RNA-dependent RNA polymerase), NSP15, NSP 16/10 (2′-O-methyltransferase), NSP 14, NSP 16, NSP 9, NSP 1, NSP 4, Orf 7a, Orf 3a, and Orf 6. The proteins with no X-ray coordinates were downloaded from the Zhang's laboratory from the University of Michigan ( https://zhanglab.ccmb.med.umich.edu/COVID-19/ ). The proteins and peptides were submitted to MolProbity webserver to check the file and fixed any potential errors in PDB (program database). The protein chains were edited for missing hydrogen atoms, bond orders, and hydrogen bonds and were optimized. The binding mode of the LL-37 peptide with all the above-listed protein targets was studied by performing peptide–protein docking using a COVID-19 docking server. 9

**Table 1:** Docking result analysis of top 7 peptide–protein complexes

Potential protein targets	Amino acids of targets interacting with LL-37 peptide			
	Binding energy (kcal/mol)	Hydrogen-bond interaction	Number of salt bridges	Nonbonded interactions
Membrane protein	−1,353.12	Asn 231; Leu 242; Asp 245; Asp 248; Asn 151; Asp 133	5	140
Nonstructural protein (Nsp 4)	−1,295.69	Asp 433, Gln 273; Asn 266; Thr 254; Arg 305; Ala 307; Asp 72; Tyr 82	2	144
Orf 3	−1,196.52	Glu 266; Thr 269; Met 5; Asp 2		
Envelope (E) protein	−1,193.21	Leu 28; Leu 39; Cys 43, Phe 4; Ser 6	**–**	90
Main protease	−1,141.8	Arg 298; Glu 107; Asp 110	4	137
Spike monomer (close)	−1,031.67	Pro 225; Glu 281; Ser 45; Lys 733	2	118
Nsp 12	−1,005.71	Asn 552; Val 166; Glu 167; Asn 437; Asp 445; Gln 444; Arg 249; Ser 451; Thr 324; Trp 268; Leu 251; Ser 255	2	234

Global docking was performed to predict the binding mode between LL-37 and target proteins using CoDockPP. The CoDockPP server performs a multistage protein–protein docking based on shape complementarity, knowledge-based scoring function, and site constraint. The best docked complex is identified based on the score value (kcal/mol) and was considered for further interaction analysis. The interaction map analysis was performed by using the PDBSum webserver ( https://www.ebi.ac.uk/thornton-srv/databases/cgi-bin/pdbsum ) and Ppor. The parameters such as number of hydrogen bonds, salt bridges, disulphide bonds, and nonbonded contacts were considered in interpreting the strength of the interaction.

The putative target for the LL-37 peptide was predicted by considering a cut-off value for binding energy score (greater than −1,000 kcal/mol). Nearly seven targets were identified with the score value above the cut-off. The membrane protein, envelope protein, main protease, spike monomer (close), NSP 4, NSP 12, and Orf 3 were identified as potential targets ( Table 1 ). Based on their binding energy and also literature sources, we predicted that the envelope and membrane proteins may be the main targets of this peptide. It is demonstrated that LL-37 was found not to alter the binding or initial uptake of the virus by cells, but regulate peptide-mediated disruption of viral membranes and further impair viral survival or propagation within the infected cells. 10

This *in silico* work also suggested some crucial information of the interaction of LL-37 with the membrane and envelope by forming hydrogen bonds, hydrophobic interactions, ionic bonds, and noncovalent bonds. It was seen the LL-37 forms hydrogen bond interactions with Asn231, Leu242, Asp245, Asp 248, Asn 151, and Asp122; hydrophobic interactions with Phe3, Trp 207, Ala 210, leu 288, and others; ionic interactions with Lys5, Glu288, Glu290, Lys12, Asp92, Glu178, and Asp155; aromatic–aromatic interactions with phe5, Tyr37, Phe103, Tyr101, Phe140, Tyr182, Tyr161, Phe159, and others; aromatic–sulfur interactions with Cys300, Phe3, Tyr 54, Phe66, Cys44, Cys22, Cys160, Cys128, Met120, Met264, and Cys262 of the membrane protein. Similarly, the residues Leu28, leu39, Cys43, Phe4, and Ser6 of the envelope protein interact with LL-37 by hydrogen bond formation. The hydrophobic interactions were formed by Met1, Tyr2, Lue21, Val22, Val58, Luu 74, and other residues. The ionic interactions between the LL37 and the envelope protein involve Arg69 and Arg72. The residues Phe23, Phe26, Phe26, and Tyr form aromatic–aromatic interactions, and Tyr2 and Cys40 are found associated with aromatic–sulfur interactions ( Table 1 ).

**Fig. 1:**
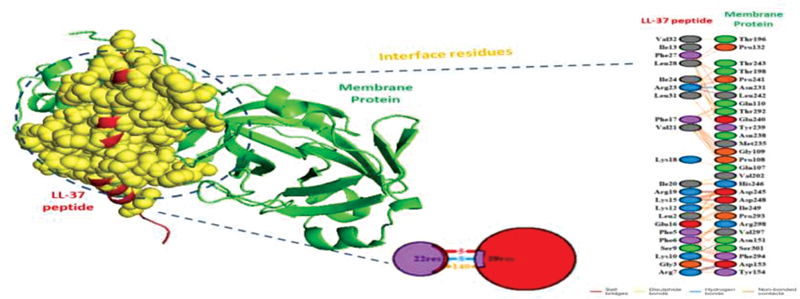
The binding mode and interactions of the LL-37 with SARS-CoV-2 membrane protein.

**Fig. 2:**
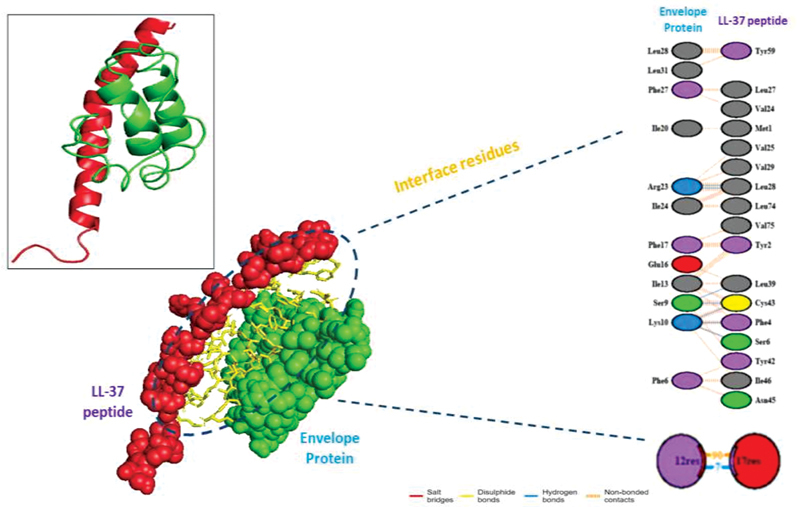
The binding mode and interactions of the LL-37 with SARS-CoV-2 envelope (E) protein.

These different types of interactions, especially hydrogen bond and hydrophobic interactions, between the LL-37 peptide and the SARS-CoV-2 membrane and envelope proteins provide a clue for potential anti-SARS-CoV-2 activity ( Figs. 1 and 2 ). Thus, it can be hypothesized that positive interfacial hydrophobicity of LL-37 increases the chance of membrane binding, i.e., interacting with the viral hydrophobic surface, and thus may kill enveloped viruses by disrupting the viral membrane and envelope, further damaging the virions in inhibiting infectivity.

## Results

The articles that analyzed various treatment protocols for reducing viral load in saliva, using various combinations, formulations, of mouthwashes in different concentrations are briefly discussed below ( Table 2 ). The various components in the mouthwashes did decrease the risk of transmission and contribute to decreased risk but did not sustain its effect for a longer period of time.

**Table 2:** Characteristics of Included studies

Author	Experimental area	Methodology	Outcome	Conclusion	Remarks
Eduardo et al, 2021	Three type of mouthwashes with solutions containing either 0.075% cetylpyridinium chloride plus 0.28% zinc lactate (CPC + Zn), 1.5% hydrogen peroxide (HP), or 0.12% chlorhexidine gluconate (CHX) were evaluated for reducing the SARS-CoV-2 viral load in saliva at different time points in this pilot randomized single-center clinical trial.	60 SARS-CoV-2-positive patients were randomly divided into a placebo (oral rinsing with distilled water) group and groups according to the type of mouthwash. Saliva samples were collected from the participants before rinsing (T0), immediately after rinsing (T1), 30 minutes after rinsing (T2), and 60 minutes after rinsing (T3). The salivary SARS-CoV-2 viral load was measured by qRT-PCR assays.	Rinsing with HP and CPC + Zn resulted in better reductions in viral load, with 15.8 ± 0.08 and 20.4 ± 3.7fold reductions at T1, respectively. Although the CPC + Zn group maintained a 2.6 ± 0.1-fold reduction at T3, this trend was not observed for HP. HP mouthwash resulted in a significant reduction in the SARS-CoV-2 viral load up to 30 minutes after rinsing (6.5 ± 3.4). The CHX mouthwash significantly reduced the viral load at T1, T2, and T3 (2.1 ± 1.5-, 6.2 ± 3.8-, and 4.2 ± 2.4-fold reductions, respectively)	Mouthwash with CPC + zinc and CHX resulted in significant reductions of the SARS-CoV-2 viral load in saliva up to 60 minutes after rinsing, while the HP mouthwash resulted in a significant reduction up to 30 minutes after rinsing.	Products could be considered as risk-mitigation strategies for patients infected with SARS-CoV-2.
Seneviratne et al, 2021	A randomized control trial in which the efficacies of three commercial mouth-rinse, viz. povidone–iodine (PI), chlorhexidine gluconate (CHX), and cetylpyridinium chloride (CPC), were assessed for the ability in reducing the salivary SARS-CoV-2 viral load in COVID-19 patients compared with water.	A total of 36 SARS-CoV-2-positive patients were recruited, of which 16 patients were randomly assigned to four groups—PI group ( *n* = 4), CHX group ( *n* = 6), CPC group ( *n* = 4), and water as the control group ( *n* = 2). Saliva samples were collected from all patients at baseline and at 5 minutes, 3 hours, and 6 hours postapplication of mouth-rinses/water. The samples were subjected to SARS-CoV-2 RT-PCR analysis.	Comparison of salivary Ct values of patients within each group of PI, CHX, CPC, and water at 5 minute, 3 hour and 6 hour time points did not show any significant differences. However, when the Ct value fold changes of each of the mouth-rinse group patients were compared with the fold change of water group patients at the respective time points, a significant increase was observed in the CPC group patients at 5 minutes and 6 hours and in the PI group patients at 6 hours.	The effect of decreasing salivary load with CPC and PI mouth-rinsing was observed to be sustained up to 6 hour time point.	This study suggests usage of cetylpyridinium chloride- and povidone–iodine-based mouthwashes for hampering viral load but it clearly says the time of presence of these components is 6 hours. Therefore, sustained release of these components for longer time might be a possible way to overcome the disadvantage.
Yoon et al, 2020	Evaluated the viral dynamics in various body fluid specimens, such as nasopharyngeal swab, oropharyngeal swab, saliva, sputum, and urine specimens, of two patients with COVID-19 from hospital day 1 to 9. Additional samples of the saliva were taken at 1 hour, 2 hours, and 4 hours after using a chlorhexidine mouthwash. The coronavirus 2 (SARS-CoV-2) viral load was determined.	SARS-CoV-2 was detected from all the five specimens of both patients by rRT-PCR.	The viral load was the highest in the nasopharynx (patient 1 = 8.41 log _10_ copies/mL; patient 2 = 7.49 log _10_ copies/mL), but it was also remarkably high in the saliva (patient 1 = 6.63 log _10_ copies/mL; patient 2 = 7.10 log _10_ copies/mL). SARS-CoV-2 was detected up to hospital day 6 (illness day 9 for patient 2) from the saliva of both patients. The viral load in the saliva decreased transiently for 2 hours after using the chlorhexidine mouthwash.	SARS-CoV-2 viral load was consistently high in the saliva; it was relatively higher than that in the oropharynx during the early stage of COVID-19. Chlorhexidine mouthwash was effective in reducing the SARS-CoV-2 viral load in the saliva for a short-term period.	The study early mentions the usage of chlorhexidine mouthwash to reduce viral load, but the only drawback is there was no comparison being made with other mouthwashes with different compositions.
Carrouel et al, 2021	To determine if commercially available mouthwash with b-cyclodextrin and citrox (bioflavonoids) (CDCM) could decrease the coronavirus 2 (SARS-CoV-2) salivary viral load	In this randomized controlled trial, severe acute respiratory syndrome coronavirus 2 (SARSCoV-2) PCR-positive patients aged 18–85 years with asymptomatic to mild coronavirus disease 2019 (COVID-19) symptoms for <8 days were recruited. A total of 176 eligible patients were randomly assigned (1:1) to CDCM or placebo. Three rinses daily were performed for 7 days. Saliva sampling was performed on day 1 at 09.00 (T1), 13.00 (T2), and 18.00 (T3). On the following 6 days, one sample was taken at 15.00. Quantitative RT-PCR was used to detect SARS-CoV-2.	The intention-to-treat analysis demonstrated that, over the course of 1 day, CDCM was significantly more effective than placebo 4 hours after the first dose ( *p* = 0.036), with a median percentage (log _10_ copies/mL) decrease T1eT2 of e12.58% (IQR: e29.55% to e0.16%). The second dose maintained the low median value for the CDCM (3.08 log _10_ copies/mL; IQR: 0e4.19), compared with placebo (3.31 log _10_ copies/mL; IQR: 1.18e4.75). At day 7, there was still a greater median percentage (log _10_ copies/mL) decrease in salivary viral load over time in the CDCM group (e58.62%; IQR: e100% to e34.36%) compared with the placebo group (e50.62%; IQR: e100% to e27.66%).	This trial supports the relevance of using CDCM on day 1 (4 hours after the initial dose) to reduce the SARS-CoV-2 viral load in saliva. For long-term effect (7 days), CDMC appears to provide a modest benefit compared with placebo in reducing viral load in saliva.	β-Cyclodextrin and citrox (bioflavonoids) (CDCM) are suggested by authors, even though other potential mouthwashes have not been used in comparison groups.
Mohebbi et al, 2021	A systematic review was conducted to determine whether mouthwashes reduce COVID-19 viral load during dental procedure and oropharyngeal examination.	A systematic search in PubMed, EMBASE, Scopus, Web of Science, and Cochrane library for relevant studies up to February 2021. Papers evaluating patients with COVID-19 infection (patients) who rinse mouthwashes (intervention) compared with patients who do not rinse them (comparison) for reducing COVID-19 viral load or reducing cross-infection of COVID-19 (outcome) in the randomized and nonrandomized clinical trials and quasi-experimental studies (study) were included due to PICOS question. Three independent authors conducted literature screening and data extraction. We extracted the most relevant data and we evaluated the risk of bias from the included studies.	Out of 344 potentially eligible articles, six studies were included in this systematic review. Regarding viral load and negative cycle threshold (ct) values, 1% PVP-I and Listerine mouthwash were effective. 0.12% CHX mouthwash was effective 0–2 hours post rinsing, but it was not effective after 2 hours. A mixture solution of 0.2% chlorhexidine gluconate and 6% hydrogen peroxide was effective on day 5 of intervention. Gargling 1% hydrogen peroxide, 0.075% CPC, 0.5%PVP-I, and 0.2% CHX mouthwashes was not effective on SARS-COV-2.	The viral load of SARS-COV-2 in saliva will be decreased after rinsing mouthwashes containing 1% PVP-I and Listerine even though not guaranteed.	The review states the limitation of mouthwashes to reduce the viral load prior to performing dental procedures and oropharyngeal examinations, there opens avenues for other strategies to decrease viral load.
Komine et al, 2021	Examined inactivation of SARS-CoV-2 by oral care products in several countries *in vitro*	0.05% cetylpyridinium chloride (CPC) mouthwash, 0.05% CPC toothpaste and 0.30% CPC spray in Japan; 0.06% chlorhexidine gluconate (CHX) + 0.05% CPC mouthwash and 0.12% CHX + 0.05% CPC mouthwash in Europe; 0.075% CPC mouthwash, 0.12% CHX mouthwash, and 0.20% delmopinol hydrochloride mouthwash in the United States; and 0.04% CPC mouthwash in China were assessed for their virucidal activity with ASTM E1052.	The virus was inactivated *in vitro* by the contact time in directions for use of all oral care products containing CPC or delmopinol hydrochloride as antiseptics.	Suggests products containing CPC or delmopinol hydrochloride as antiseptics decrease viral loads.	Comparisons done are clearer and results clearly suggest usage of CPC or delmopinol hydrochloride-based products

Abbreviation: RT-PCR, reverse transcription polymerase chain reaction.

## Discussion

### Strategies to Tackle SARS-CoV-2 Viral Load in Saliva

Salivary viral load of SARS-CoV-2 is claimed by World Health Organization to be a major transmission factor because it is the entry of the respiratory system and also has been tested for presence of 2019-nCoV nucleic acid in salivary samples collected from infected individuals. Various studies mention the load of COVID-19 virus in saliva can be used to correlate with the severity of disease state. 11 Comparison of patient demographics, cellular immune profiling data with salivary and nasopharyngeal (NP) swabs by estimating the viral load over time, was conducted among 154 SARS-CoV-2 reverse transcription quantitative polymerase chain reaction (RT-q-PCR)-positive eligible adults and 109 uninfected HCWs. The viral RNA, SARS-CoV-2 specific-antibody, cytokine levels, platelets, lymphocytes, and cTfh cell kinetics were compared between the groups. A total of 154 SARS-CoV-2 RT-q-PCR-positive adults were included in the study group; 109 uninfected HCWs were included in this study as the control group. Individuals with SARS-CoV-2 risk factors showed a higher load of virus in saliva which could be correlated with the increasing disease severity, which could be attributed as a predictor of mortality over time (area under the curve = 0.90). 12 The potential of saliva transmission of COVID-19 can be due to the cell receptor of ACE2 expression in the tongue and salivary glands. Thus, it is important to prevent droplet formation and transmission. 1 Saliva harbors various antiviral components like lysozyme, mucins, cathelicidin (LL-37), lactoferrin, peroxidase, sIgA SLPI, salivary agglutinin (gp340, DMBT1), α-defensins, β-defensins, and cystatins, which hamper viral multiplication. The microvesicles with 20 microRNAs restrict multiplication of viruses, but the major possible factor could be hypo-salivation which causes increased multiplication and adhesion of virus due to decreased salivary proteins and peptides. 13 An oral axis for SARS-CoV-2 pathogen and transmission was planned to evaluate the expression of SARS-CoV-2 entry into cells of salivary glands. The mode of asymptomatic transmission of the virus remains “Achilles heel” in this pandemic situation. Due to anatomical location, frequent exposure to external environment, oral tissues, and saliva may play a major factor in the spread of SARS-CoV-2. 14

It is documented through various studies that salivary viral load of SARS-CoV-2 is diagnostic in nature, which helps in correlating with the disease severity, plays a role in transmission of disease, and therefore strategies to tackle and reduce the viral load play a prime role. 15 A collective opinion from Various studies provided a collective opinion to use 0.2% povidone iodine(PI) for 30 sec as a pre procedural rinse followed by gargles for 30 seconds and throat gargle for 1minute in dental clinics which may act as a potent decontaminating agent in the reduction of SARS-COV-2 virus in the oral cavity. D'Amico et al suggested 1% solution of hydrogen peroxide usage for 30 seconds with 0.2 to 0.03% chlorhexidine to reduce viral/bacterial load; components of chlorhexidine are considered to be effective against lipid enveloped viruses; 0.12% 5 mL of chlorhexidine could suppress viral loads for 2 hours, even though it was beneficial in hampering droplet transmission in some patients, did not reduce salivary viral load in many after 1 hour of use. 16 CPC (cetylpyridinium chloride), a quaternary ammonia salt in a concentration of 0.02 to 0.07% for oral use, provided a detergent action and demonstrated disruption of lipid bilayer envelope of SARS-CoV-2 virus. It is also one among the most recommended mouthwashes for use in SARS -COV-2 patients. 17 Mouthwash with CPC + zinc and CHX resulted in significant reductions of the SARS-CoV-2 viral load in saliva up to 60 minutes after rinsing, while hydrogen peroxide mouthwash resulted in a significant reduction up to 30 minutes after rinsing. 18 In a randomized control trial in which the efficacy of three commercial mouth-rinses were evaluated, povidone–iodine (PI), chlorhexidine gluconate (CHX), and CPC; CPC decreased salivary load of SARS-CoV-2 and PI mouth-rinsing was observed to sustain up to 6-hour time point. 19 Stawarz-Janeczek et al recommended effective usage of 0.1% sodium hypochlorite, 0.5% hydrogen peroxide, ethanol at the minimum concentration of 62 to 71% in 1 minute to reduce viral load. Ethanol at a concentration of 78 to 95% for 30 sec to 1 min reduced salivary viral load of SARS COV-2 drastically. 20 Also various oral care products were assessed and further *in vitro* studies among 0.5% CPC mouthwash, 0.05% CPC toothpaste and 0.30% CPC spray, 0.06% CHX + 0.05% CPC mouthwash, 0.12% CHX + 0.05% CPC mouthwash, 0.075% CPC mouthwash, 0.12% CHX mouthwash, 0.20% delmopinol hydrochloride mouthwash and 0.04% CPC mouthwash were evaluated for their antiviral activity. Suggested products containing CPC or delmopinol hydrochloride as antiseptics decrease viral loads. 20 Farook et al recommend preprocedural rinse with chlorhexidine, PI, which is being used in dental clinics to reduce viral load in oral cavity even through it is not established, 21 either with usage of 0.12% of chlorhexidine, 1% of hydrogen peroxide or 0.2% of PI solution. 22 23 LL-37 is proved through *in silico* work to have capacity to disrupt the viral membrane; various studies have employed the AMP LL-37 in different treatment strategies in various viral conditions with therapeutic effect.

### Potential of LL-37 Peptide to Inhibit Viral Infections

Various authors and researchers have explained the potential of LL-37 in tackling various viral conditions. I *n vitro* experiments and *in silico* analyses have shown that LL-37 inhibits dengue virus type 2 at the stage of entry into the cells by binding to the E protein, thus might be potent against dengue virus infections. 24 Therapeutic activity of LL-37 against influenza type A virus (IAV) demonstrates LL-37 encounters IAV in the respiratory tract through innate immune responses against the virus, secreted through neutrophils, macrophages, and epithelial cells. The study further concluded that LL-37 reduced IAV infection severity in a manner comparable to zanamivir. 25 LL-37 has shown inhibition of HIV 1 protease activity, furthermore the plasma LL-37 levels of individuals undergoing antiretroviral therapy (ART) were significantly higher, in contrast, high susceptibility to secondary infections was observed in patients not undergoing ART. 26 The efficacy of LL-37 peptide against RSV was demonstrated in a study which clearly showed that this peptide had potential to inhibit viral-induced cell death, impact the expression of chemokines, and showed that children with low cathelicidin levels are more susceptible to RSV-associated bronchitis. 27 A study showed the effect of LL-37 AMP on decreasing viral load of HRV, a causative factor for common cold and most respiratory infections. The study concluded the LL-37 expression decreased HRV viral load *in vivo* and thereby decreases infections in respiratory cells and cystic fibrosis cells. 28 LL-37 was delivered through corneal implants and assessment of release was done, which showed that even though LL-37 did not block the herpes simplex virus 1 (HSV-1) action it prevented infection in corneal epithelial cells by preventing viral cell attachment, hence proving that LL-37 actively takes part in entry inhibition. 29 Zika, a single-stranded RNA virus, is responsible for “microcephaly ventriculomegaly intracranial infectious disease” related to birth defects; a study was conducted to evaluate the effect of LL-37 and synthetic derivatives on primary human fetal astrocytes. The study showed sevenfold decrease in ZIKV plaque-forming unit's posttreatment, with LL-37 *in vitro* . 30 The host defense peptide LL-37 was studied against VEEV, and data showed that LL-37 exhibits robust antiviral activity with minimal toxicity to humans, and blocks the virus from entering human cells. 6 A study discussed the effect of LL-37 in combating rhinovirus and further concluded that delivery of LL -37 has novel synthetic analogues representing a novel treatment module for reducing the viral load. 31 The effect of LL-37 on inhibition of Ebola virus (EBOV) was studied, with regard to the outbreak of Ebola epidemic in South Africa in the period of 2014 to 2016.Various therapeutic modalities were tested and showed that human cathelicidin AMP LL-37 and engineered LL-37 AMPs inhibit the infection of recombinant virus pseudo-typed with EBOV glycoprotein (GP) and the wild-type EBOV. These AMPs target EBOV infection at the endosomal cell-entry step by impairing cathepsin B-mediated processing of EBOV GP. 31 Results identify AMPs as a novel class of anti-EBOV therapeutics and demonstrate the feasibility of engineering AMPs for improved therapeutic efficacy. 31 Ron-Doitch et al prepared LL-37 liposomes coated with PEG and evaluated their activity against HSV-1. They found lower toxicity and enhanced antiviral activity for LL-37 liposomes compared with both the free AMPs. 32 Nordström et al investigated how the charge density in poly(ethyl acrylate-co-methacrylic acid) or poly (ethyl acrylate/methacrylic acid [MAA]/1,4-butandiol diacrylate) microgels (MAA26.5 and MAA60 microgels) affects the capacity to release the peptides LL-37 effective against *Pseudomonas aeruginos* a and *Escherichia coli* bacteria, and studied hemolysis, proteolytic stability, and interaction of loaded hydrogel with membranes. 33 Fan et al conducted a study to detect the efficiency and antitumor effect of docetaxel and LL-37-loaded thermosensitive hydrogel nanoparticles on peritoneal carcinomatosis of colorectal cancer. They concluded that the Doc + LL37 NP–hydrogel composite showed improved antiangiogenesis and antitumor activity, and thus may have potential applications in colorectal carcinoma therapy. 34 Fumakia and Ho investigated the potential therapeutic effects of combining LL37 and A1 into a single nanoparticle formulation to improve wound healing and synergistically enhance antibacterial activity *in vitro* , in comparison to individual drugs alone, and concluded that they successfully developed the first solid lipid nanoparticle formulation that can simultaneously deliver LL37 and A1 at specific ratios resulting in accelerated wound healing by promoting wound closure in BJ fibroblast cells and keratinocytes as well as synergistically enhancing antibacterial activity against *Staphylococcus aureus* and *E. coli* in comparison to LL37 or A1 alone. 35

## Conclusion

The analysis of docking studies and the display of positive interfacial hydrophobicity of LL-37 resulting in disruption of COVID-19 viral membrane elucidate the fact that LL-37 could be effective against all variants of SARS-CoV-2. Further experimental studies would be needed to confirm the binding of RBD with LL-37. Its immunoregulatory pathway would be at a faster pace when compared with different therapeutics that being researched and developed, owing to its nativity to the human body. The article provides the potential of LL-37 in combating SARS-CoV-2 in saliva. It can be employed strategically in different ways, which will help in mitigating risk to frontline workers, who come in contact with patients in COVID wards, and dentists who are in danger because they are exposed to oral cavity. The current article is an attempt to showcase the therapeutic potential of LL-37. The possibility of using it in many forms further to decrease the viral load by disrupting the viral membrane is seen.

## References

[1] Xu R Cui B Duan X Zhang P Zhou X Yuan Q Saliva: potential diagnostic value and transmission of 2019-nCoV Int J Oral Sci 2020 12 01 11 32300101 10.1038/s41368-020-0080-z PMC7162686

[2] Guo Y R Cao Q D Hong Z S The origin, transmission and clinical therapies on coronavirus disease 2019 (COVID-19) outbreak - an update on the status Mil Med Res 2020 7 01 11 32169119 10.1186/s40779-020-00240-0 PMC7068984

[3] Liu Q Luo D Haase J E The experiences of health-care providers during the COVID-19 crisis in China: a qualitative study Lancet Glob Health 2020 8 06 e790 e798 32573443 10.1016/S2214-109X(20)30204-7 PMC7190296

[4] Diamond G Beckloff N Weinberg A Kisich K O The roles of antimicrobial peptides in innate host defense Curr Pharm Des 2009 15 21 2377 2392 19601838 10.2174/138161209788682325 PMC2750833

[5] Pahar B Madonna S Das A Albanesi C Girolomoni G Immunomodulatory role of the antimicrobial LL-37 peptide in autoimmune diseases and viral infections Vaccines (Basel) 2020 8 03 517 10.3390/vaccines8030517 PMC7565865 32927756

[6] Ahmed A Siman-Tov G Hall G Bhalla N Narayanan A Human antimicrobial peptides as therapeutics for viral infections Viruses 2019 11 08 704 10.3390/v11080704 PMC6722670 31374901

[7] Barlow P G Findlay E G Currie S M Davidson D J Antiviral potential of cathelicidins Future Microbiol 2014 9 01 55 73 24328381 10.2217/fmb.13.135

[8] Mookherjee N Hancock R E Cationic host defence peptides: innate immune regulatory peptides as a novel approach for treating infections Cell Mol Life Sci 2007 64(7-8):922 933 17310278 10.1007/s00018-007-6475-6 PMC11136131

[9] Kong R Yang G B Xue R COVID-19 docking server: a meta server for docking small molecules, peptides and antibodies against potential targets of COVID-19 2020 36 20 5109 5111 10.1093/bioinformatics/btaa645 PMC7558834 32692801

[10] Tripathi S Tecle T Verma A Crouch E White M Hartshorn K L The human cathelicidin LL-37 inhibits influenza A viruses through a mechanism distinct from that of surfactant protein D or defensins J Gen Virol 2013 94(Pt 1):40 49 23052388 10.1099/vir.0.045013-0 PMC3542722

[11] Amorim Dos Santos J Normando A GC Carvalho da Silva R L Oral manifestations in patients with COVID-19: a living systematic review J Dent Res 2021 100 02 141 154 32914677 10.1177/0022034520957289

[12] Silva J Lucas C Sundaram M Saliva viral load is a dynamic unifying correlate of COVID-19 severity and mortality medRxiv 2021 10.1101/2021.01.04.21249236

[13] Baghizadeh Fini M Oral saliva and COVID-19 Oral Oncol 2020 108 104821 32474389 10.1016/j.oraloncology.2020.104821 PMC7250788

[14] NIH COVID-19 Autopsy Consortium HCA Oral and Craniofacial Biological Network Huang N Pérez P Kato T SARS-CoV-2 infection of the oral cavity and saliva Nat Med 2021 27 05 892 903 33767405 10.1038/s41591-021-01296-8 PMC8240394

[15] To W-K Tsang O T Yip C C Consistent detection of 2019 novel coronavirus in saliva Clin Infect Dis 2020 71 15 841 843 32047895 10.1093/cid/ciaa149 PMC7108139

[16] D'Amico C Bocchieri S Stefano R Dental office prevention of coronavirus infection Eur J Dent 2020 14(S 01):S146 S151 33285574 10.1055/s-0040-1715923 PMC7775218

[17] Eduardo F P Corrêa L Heller D Salivary SARS-CoV-2 load reduction with mouthwash use: a randomized pilot clinical trial Heliyon 2021 7 06 e07346 34189331 10.1016/j.heliyon.2021.e07346 PMC8222261

[18] Seneviratne C J Balan P Ko K KK Efficacy of commercial mouth-rinses on SARS-CoV-2 viral load in saliva: randomized control trial in Singapore Infection 2021 49 02 305 311 33315181 10.1007/s15010-020-01563-9 PMC7734110

[19] Yoon J G Yoon J Song J Y Clinical significance of a high SARS-CoV-2 viral load in the saliva J Korean Med Sci 2020 35 20 e195doi:10.3346/jkms.2020.35.e195 32449329 10.3346/jkms.2020.35.e195 PMC7246183

[20] Carrouel F Valette M Gadea E Use of an antiviral mouthwash as a barrier measure in the SARS-CoV-2 transmission in adults with asymptomatic to mild COVID-19: a multicentre, randomized, double-blind controlled trial Clin Microbiol Infect 2021 27 10 1494 1501 34044151 10.1016/j.cmi.2021.05.028 PMC8142805

[21] Mohebbi Z Ebrahimi S T Shamshiri A R Do mouthwashes reduce Covid-19 viral load during dental procedures and oropharyngeal examinations? A systematic review Preprints 2021 10.20944/preprints202106.0249.v1still in preprint

[22] Komine A Yamaguchi E Okamoto N Yamamoto K Virucidal activity of oral care products against SARS-CoV-2 in vitro J Oral Maxillofac Surg Med Pathol 2021 33 04 475 477 33643836 10.1016/j.ajoms.2021.02.002 PMC7898974

[23] Stawarz-Janeczek M Kryczyk-Poprawa A Muszyńska B Opoka W Pytko-Polończyk J Disinfectants used in stomatology and SARS-CoV-2 infection Eur J Dent 2021 15 02 388 400 33694135 10.1055/s-0041-1724154 PMC8184310

[24] Farook F F Mohamed Nuzaim M N Taha Ababneh K Alshammari A Alkadi L COVID-19 pandemic: oral health challenges and recommendations Eur J Dent 2020 14(S 01):S165 S170 33233004 10.1055/s-0040-1718641 PMC7775230

[25] Melo Neto C LM Bannwart L C de Melo Moreno A L Goiato M C SARS-CoV-2 and dentistry-review Eur J Dent 2020 14(S 01):S130 S139 32932534 10.1055/s-0040-1716438 PMC7775231

[26] Tripathi S Wang G White M Qi L Taubenberger J Hartshorn K L Antiviral activity of the human cathelicidin, LL-37, and derived peptides on seasonal and pandemic influenza A viruses PLoS One 2015 10 04 e0124706 25909853 10.1371/journal.pone.0124706 PMC4409069

[27] Ahmed A Siman-Tov G Keck F Human cathelicidin peptide LL-37 as a therapeutic antiviral targeting Venezuelan equine encephalitis virus infections Antiviral Res 2019 164 61 69 30738837 10.1016/j.antiviral.2019.02.002

[28] Mansbach J M Piedra P A Borregaard N Serum cathelicidin level is associated with viral etiology and severity of bronchiolitis J Allergy Clin Immunol 2012 130 04 10070 10080 10.1016/j.jaci.2012.07.044 PMC3462235 22944482

[29] Schögler A Muster R J Kieninger E Vitamin D represses rhinovirus replication in cystic fibrosis cells by inducing LL-37 Eur Respir J 2016 47 02 520 530 26585423 10.1183/13993003.00665-2015

[30] Lee C J Buznyk O Kuffova L Cathelicidin LL-37 and HSV-1 corneal infection: peptide versus gene therapy Transl Vis Sci Technol 2014 3 03 4 10.1167/tvst.3.3.4 PMC4043105 24932432

[31] He M Zhang H Li Y Cathelicidin-derived antimicrobial peptides inhibit Zika virus through direct inactivation and interferon pathway Front Immunol 2018 9 722 29706959 10.3389/fimmu.2018.00722 PMC5906549

[32] Sousa F H Casanova V Findlay F Cathelicidins display conserved direct antiviral activity towards rhinovirus Peptides 2017 95 76 83 28764966 10.1016/j.peptides.2017.07.013 PMC5577862

[33] Yu Y Cooper C L Wang G Engineered human cathelicidin antimicrobial peptides inhibit Ebola virus infection iScience 2020 23 04 100999 32252021 10.1016/j.isci.2020.100999 PMC7104201

[34] Ron-Doitch S Sawodny B Kühbacher A Reduced cytotoxicity and enhanced bioactivity of cationic antimicrobial peptides liposomes in cell cultures and 3D epidermis model against HSV J Control Release 2016 229 163 171 27012977 10.1016/j.jconrel.2016.03.025

[35] Nordström R Nyström L Andrén O CJ Membrane interactions of microgels as carriers of antimicrobial peptides J Colloid Interface Sci 2018 513 141 150 29145017 10.1016/j.jcis.2017.11.014

[36] Fan R Tong A Li X Enhanced antitumor effects by docetaxel/LL37-loaded thermosensitive hydrogel nanoparticles in peritoneal carcinomatosis of colorectal cancer Int J Nanomedicine 2015 10 7291 7305 26664119 10.2147/IJN.S89066 PMC4672756

[37] Fumakia M Ho E A Nanoparticles encapsulated with LL37 and serpin A1 promotes wound healing and synergistically enhances antibacterial activity Mol Pharm 2016 13 07 2318 2331 27182713 10.1021/acs.molpharmaceut.6b00099

